# cDNA-AFLP analysis reveals differential gene expression in incompatible interaction between infected non-heading Chinese cabbage and *Hyaloperonospora parasitica*

**DOI:** 10.1038/hortres.2016.34

**Published:** 2016-07-27

**Authors:** Dong Xiao, Shi-Tuo Liu, Yan-Ping Wei, Dao-Yun Zhou, Xi-Lin Hou, Ying Li, Chun-Mei Hu

**Affiliations:** 1 Horticulture Department, College of Horticulture, Nanjing Agricultural University, Nanjing 210095, China; 2 State Key Laboratory of Crop Genetics & Germplasm Enhancement, Ministry of Agriculture, Nanjing 210095, China

## Abstract

Non-heading Chinese cabbage (*Brassica rapa* ssp. *chinensis*) is one of the main green leafy vegetables in the world, especially in China, with significant economic value. *Hyaloperonospora parasitica* is a fungal pathogen responsible for causing downy mildew disease in Chinese cabbage, which greatly affects its production. The objective of this study was to identify transcriptionally regulated genes during incompatible interactions between non-heading Chinese cabbage and *H. parasitica* using complementary DNA-amplified fragment length polymorphism (cDNA-AFLP). We obtained 129 reliable differential transcript-derived fragments (TDFs) in a resistant line ‘Suzhou Qing’. Among them, 121 upregulated TDFs displayed an expression peak at 24–48 h post inoculation (h.p.i.). Fifteen genes were further selected for validation of cDNA-AFLP expression patterns using quantitative reverse transcription PCR. Results confirmed the altered expression patterns of 13 genes (86.7%) revealed by the cDNA-AFLP. We identified four TDFs related to fungal resistance among the 15 TDFs. Furthermore, comparative analysis of four TDFs between resistant line ‘Suzhou Qing’ and susceptible line ‘Aijiao Huang’ showed that transcript levels of TDF14 (*BcLIK1*_A01) peaked at 48 h.p.i. and 25.1-fold increased in the resistant line compared with the susceptible line. Similarly, transcript levels of the other three genes, TDF42 (*BcCAT3*_A07), TDF75 (*BcAAE3*_A06) and TDF88 (*BcAMT2*_A05) peaked at 24, 48 and 24 h.p.i. with 25.1-, 100- and 15.8-fold increases, respectively. The results suggested that the resistance genes tended to transcribe at higher levels in the resistance line than in the susceptible line, which may provide resistance against pathogen infections. The present study might facilitate elucidating the molecular basis of the infection process and identifying candidate genes for resistance improvement of susceptible cultivars.

## Introduction

It is well known that plant–pathogen interactions activate a subset of pathogen genes so-called systemic acquired resistance to protect themselves.^1–3^ This interaction process is diverse and complicated because plant pathogens have evolved by developing various strategies to infect their hosts. Specific pathogen may trigger defense systems that are essential for pathogenicity. Next, molecular responses are up- or downregulations by numerous specific resistant genes. During the development of interaction, the recognition of specific host genes determines whether the interaction will be successful.

Downy mildew is an important fungal disease of *Brassica* specie that is caused by the obligatory biotrophic oomycete *Hyaloperonospora parasitica* (formerly *Peronospora parasitica* (Pers. Ex Fr.)), and infects most members of the *Brassica* family.^4^ It can be fatal to seedling growth in the nurseries and reduce the productivity and quality of adult plants in the field.^4^ Leaves become yellow after infection and then scorch. When downy mildew became epidemic, it can cause damage to >90% of the crop. The disease is more severe in spring and autumn seasons than in other seasons. Currently, the downy mildew disease is controlled by application of fungicides.^5^ However, chemical control is often difficult and ineffective. It has been proved that the most efficient way to manage plant diseases is to develop a host resistance in new cultivars.^5^ Therefore, to identify the host resistant genes is a crucial need for obtaining reliable resistant genotypes to assist plant breeding. Previous studies have shown some differentially expressed genes during infection process using various methods.^6,7^ The differential display-based strategy has been used to reveal genes related to downy mildew infection in *B. oleracea* seedlings.^6^ Suppression subtractive hybridization technology has been employed and revealed 37 high-quality Expressed Sequence Tags (ESTs), of which functions are known in energy metabolism, transcriptional regulation, signal transduction and defense reaction.^7^ However, most molecular components of the signal transduction pathway involved in gene regulation remain to be identified.

Furthermore, there is no report on pathogen virulence genes matching the resistance genes of non-heading Chinese cabbage (*Brassica rapa* ssp. *chinensis*), and their inheritance remains uncertain. In this prospect, it is important to elucidate the molecular mechanisms or gene expression profile and to identify an inventory of candidate genes during the non-heading Chinese cabbage–*H. parasitica* interaction.

Screening for differentially expressed genes is a direct approach to reveal the molecular basis of a biological system. The complementary DNA-amplified fragment length polymorphism (cDNA-AFLP) method has been successfully used for the identification of genes involved in various plant–pathogen systems.^8,9^ In comparison with microarray technique and RNA sequencing, cDNA-AFLP costs less and does not require sequence information. When compared with subtractive hybridization, cDNA-AFLP is highly reproducible.^8^

The objectives of this study were to apply the cDNA-AFLP technique to the pathogenic interaction between non-heading Chinese cabbage and downy mildew. We identified a set of genes that were regulated during the incompatible interaction between the host and pathogen, and validated the expression patterns for the regulated genes.

## Materials and methods

### Plant material, inoculums and pathogen infection

Two non-heading Chinese cabbage inbred lines, ‘Suzhou Qing’ (resistant to *H. parasitica*) and ‘Aijiao Huang’ (susceptible to *H. parasitica*) from our lab, were used in this study. The transcript-derived fragments (TDFs) were obtained from interaction between ‘Suzhou Qing’ and downy mildew (*H. parasitica*). ‘Aijiao Huang’ was used for the comparison of expression patterns of the four genes related with fungal resistance between resistant and susceptible line.

Plants were grown in plastic nurseries (inner size: 45×45 mm; height: 57 mm) and transferred to a growth chamber under 25 °C day/ 20 °C night temperature with 85±5% relative humidity and a 12-h light/12-h dark after germination for 36 h under dark. *H. parasitica* was isolated from leaves of susceptible line ‘Aijiao Huang’ in the Jiangpu Farm of Nanjing Agricultural University, China.^10^ Conidial suspensions were adjusted to 1×10^5^ spores per mL and Tween-20 was added as a surfactant to a final concentration of 0.1%. One hundred of 3-week-old seedlings (with four true-leaves) were sprayed with 50 mL pathogen suspension and distilled water (as control), respectively. After inoculation, the seedlings were covered with plastic film separately and transferred to a growth chamber under 20 °C, 100% relative humidity in the dark for the first 24 h to promote sporulation, then moved back to the initiatory conditions. Both control and treated third leaf of five plants were harvested and pooled at 0, 24, 48 and 72 h post inoculation (h.p.i.), immediately frozen in liquid nitrogen and stored at −70 °C until use.

### RNA isolation and cDNA-AFLP analysis

Total RNAs were extracted using the RNAeasy Plant Mini kit (Qiagen; https://www.qiagen.com/cn/shop/sample-technologies/rna/rna-preparation/rneasy-mini-kit#orderinginformation) and synthesis of the first strand of cDNA is made using the M-MLV reverse transcriptase (Takara Shuzo Co., Ltd, Japan) according to the manufacturer’s protocol. To synthesize the second strand, the following components were added to the first-strand solution. A volume of 30 μL, 5×2nd strand synthesis buffer, 3 μL dNTP mixture, 89 μL RNase-free H_2_O, 2 μL *Escherichia coli* DNA polymerase I, 2 μL *E. coli *RNase H*/E. coli* DNA ligase mixture and 4 μL T_4_ DNA polymerase in a final volume of 150 μL. The components were gently mixed and incubated at 16 °C for 2 h. Double-stranded cDNA was purified using the DNA Fragment Purification Kit Ver.2.0 (Takara).

Second-strand cDNA was digested by two restriction enzymes *Tag І* (restriction site TCGA) and *Ase І* (restriction site ATTAAT; Takara), and then ligated to *Tag І* and *Ase І* double-strand adaptors. The AFLP adaptor primers 5′-GACGATGAGTCCTGAC-3′, 5′-CGGTCAGGACTCAT-3′ (*Taq I*-adaptor primers) and 5′-GCGTAGACTGCGTACC-3′, 5′-TAGGTACGCAGTC-3′ (*Ase І*-adaptor primers) were ligated onto the restriction fragments: *Taq I* pre-amplification primer, 5′-GACGATGAGTCCTGACCGA-3′; *Ase I* pre-amplification primer, 5-CTCGTAGACTGCGTACCTAAT-3′; *Taq I* selective amplification primer, 5′-GATGAGTCCAGACCGA+NN-3′; *Ase I* selective amplification primer, 5′-GACTGCGTACCTAAT+NN-3′ (indicated by N, representing an A, C, G or T). The initial small-scale screen using 96 AFLP primer combinations were done using six *Taq I* forward selective amplification primers (extension CG, CA, CT, CC, GA or GT) in combination with 16 *Ase I* reverse selective amplification primers (extension NN), respectively. Pre-amplification PCR was carried out with one-tenth volume of the restriction/ligation mix, the pre-amplification PCR was carried out as follows: 94 °C, 3 min; 94 °C, 30 s, 55 °C, 30 s, 72 °C, 60 s, 25 cycles; and 72 °C, 5 min. The products of pre-amplification was diluted 10-fold, and the selective amplification PCR was carried out as follows: 94 °C, 30 s; 94 °C, 30 s, 65 °C, 30 s (−0.7 °C per cycle), 72 °C, 60 s, 12 cycles; 94 °C, 30 s, 56 °C, 30 s, 72 °C, 60 s, 24 cycles; and 72 °C, 5 min.

Selective amplification products were separated on a 6% polyacrylamide gels running at 60 W for 2 h and visualized by silver staining. Differential bands were excised from the polyacrylamide gel electrophoresis gels based on the alignment between films and markers on the gels, and incubated in 30 μL of water and then at 95 °C for 30 min. The TDFs were then re-amplified by PCR using same primers under the similar conditions. The amplified fragments were retrieved from a 1% agarose gel with the Sephaglas BandPrep kit (Amersham Pharmacia Biotech.), cloned into pGEM-T Easy vector (Takara) according to the manufacturer's protocol and sequenced by Invitrogen Company (Shanghai BioWisdom Technology Co, Ltd; Shanghai, China. http://en.cellfood.com.cn/culture.aspx), and sequence information was BLASTed in the *Brassica* database (http://*brassica*db.org/brad/).

### Quantitative real-time PCR

The single-strand cDNA of resistant line ‘Suzhou Qing’ and susceptible line ‘Aijiao Huang’ were diluted to 30 ng μL^−1^, and were used for quantitative reverse transcription PCR (qRT-PCR) analyses. Primers were designed by the Primers 3 (http://frodo.wi.mit.edu/primer3/) based on the interested cDNA sequence. The qRT-PCR reaction mixtures contained 12.5 μL, 2× SYBR Green PCR MasterMix (Applied Biosystems; http://www.bio-rad.com/), 10 pm of each primer, 2 μL template and sterile distilled water to total volume of 25 μL, as well as also performed on CFX96 Real-Time System (C1000 Thermal Cycler, Bio-Rad, CA, USA). Thermal conditions were 2 min of denaturation at 95 °C, followed by 45 cycles of 95 °C for 10 s, annealing at 55 °C for 20 s, and extension at 72 °C for 20 s and 72 °C for 5 min. Three technical replicates were analysed for each biological replicate. All the cycle threshold (Ct) values from one gene were determined at the same threshold fluorescence value of 0.2 using the ΔΔCt method.^11^ The primers of gene-specific and housekeeping gene were listed in Table 1.

Statistical analysis was performed using Student’s *t*-test.

## Results

### Isolation of differentially expressed genes

**Figure 1:**
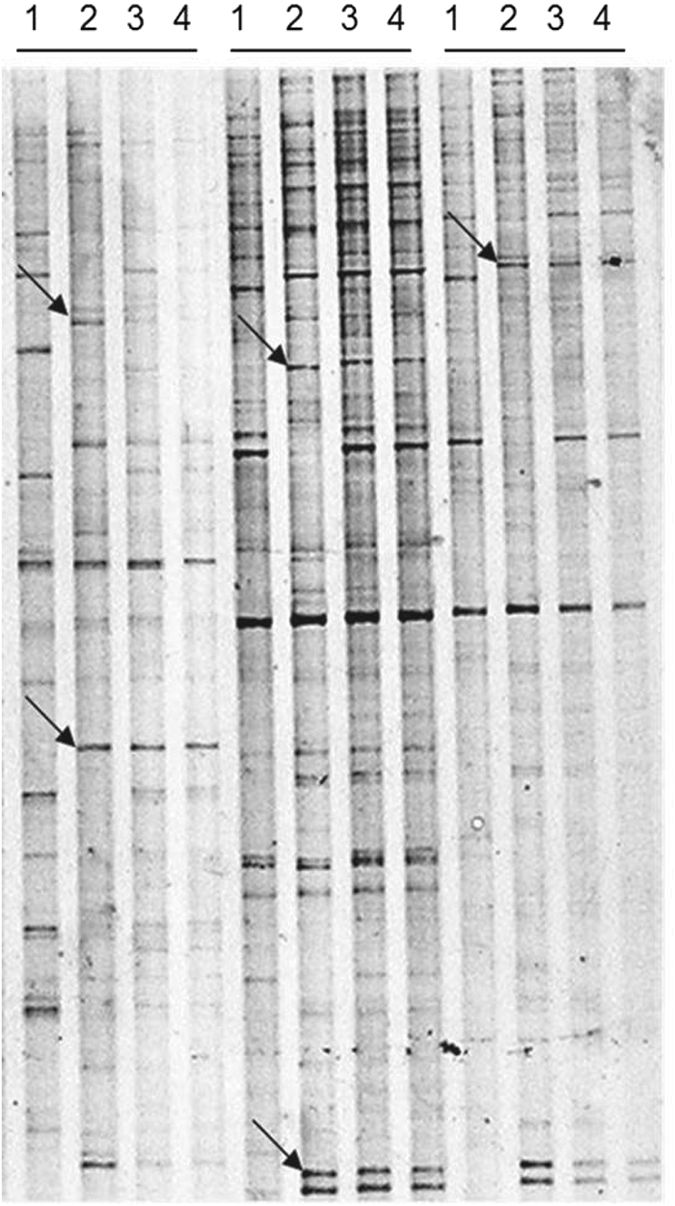
Expression of non-heading Chinese cabbage ‘Suzhou Qing’ genes in leaves inoculated with *H. parasitica* transcripts was displayed by cDNA-AFLP. An example showing that land 1, 2, 3 and 4 represents the induction time 0, 24, 48 and 72 h.p.i., respectively. The size of the differential TDFs was determined by direct sequencing. Arrow: differential bands.

To determine the early events involved during the non-heading Chinese cabbage–*H. parasitica* interactions, four gene pools were constructed from resistant inbred line ‘Suzhou Qing’ at 0, 24, 48 and 72 h.p.i., respectively. TDFs displayed by cDNA-AFLP analysis ranged in size from 100 to 800 bp, depending on 96 selective primer combinations and time points. Figure 1 showed an example of the expression patterns of the genes revealed using cDNA-AFLP. A total of 180 fragments were obtained with the 96 primer pairs. After excluding repeat and error sequences, 129 TDFs were obtained. Of the 129 TDFs, 121 were upregulated and 8 downregulated. Of the 121 TDFs upregulated, 35 (28.9%), 31 (25.6%) and 4 (3.3%) TDFs were induced strongly at 24, 48 and 72 h.p.i., respectively; 12 (9.9%) and 2 (1.7%) TDFs were induced at 24 and 48 h.p.i., and 48 and 72 h.p.i., respectively. These results showed that non-heading Chinese cabbage has mainly accumulated expression at 24–48 h.p.i. and that gene expression patterns were different and complex after *H. parasitica* infection.

### Gene sequence analysis

**Figure 2:**
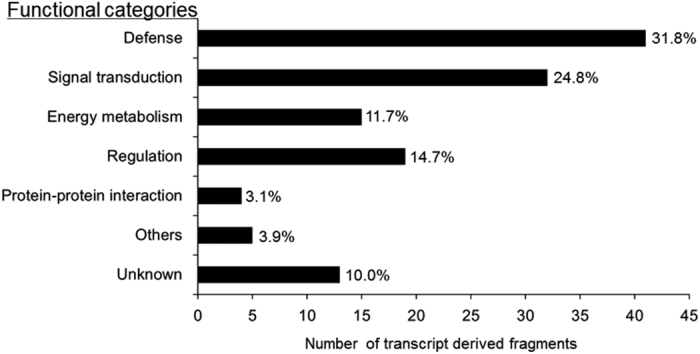
Classification of differentially accumulated TDFs after inoculation of *H. parasitica*. A total of 129 TDFs were classified based on the Chinese cabbage database.

By BLASTn search on *Brassica* database, 129 TDFs were successfully annotated (Table 2). One hundred and sixteen TDFs (90%) of the 129 TDFs can be divided into six functional categories, including defense (D), signal transduction (ST), energy metabolism (EM), regulation (R), protein–protein interaction (PI), others (O) and unknown (Un; Figure 2). Forty-one TDFs (31.8%) of the annotated sequences were associated with defense. Among them, four TDFs were associated with the pathogenesis-related protein, include *β-1,3-glucanase* (TDF3), *hapless 8* (TDF8), *pathogenesis-related protein* (TDF28) and *thaumatin-like protein* (TDF16), and others had *hypersensitive-induced response protein* (TDF22), *mannose-binding lectin superfamily protein* (TDF23), *respiratory burst oxidase protein* (TDF13) and so on. Thirty-two TDFs (24.8%) were involved in signal transduction, for example, a member of the *BEL family of homeodomain proteins* (TDF49), *catalase* (TDF42), *calcium ion binding *(TDF48) and so on. Followed that, 19 TDFs (14.7%) mainly involved in regulation, including *ATP-binding cassette G36* (TDF91), *heat-shock cognate protein* (TDF102) and so on. Fifteen TDFs (11.7%) were mainly involved in energy metabolism, for example, TDF81 was predicted to involve in *Arabidopsis thaliana* photosynthetic electron transfer chain. Four TDFs (3.1%) and five TDFs (3.9%) were involved in protein–protein interaction and others metabolic pathways, respectively. These genes might function to protect cells from the fungal pathogen in non-heading Chinese cabbage. No function was assigned to 13 (10.0%) of the TDFs as they showed no or low sequence similarities in the *Brassica* database search. In conclusion, gene expression patterns are more complex after infection and involved in many different metabolic pathways. It indicates that it is the common effect of these different metabolic pathways that improved the plant resistance to fungus, thereby reducing the hypersensitive response (HR) in resistance line against fungus pathogen.

### Validation of expression patterns using qRT-PCR analysis

**Figure 3:**
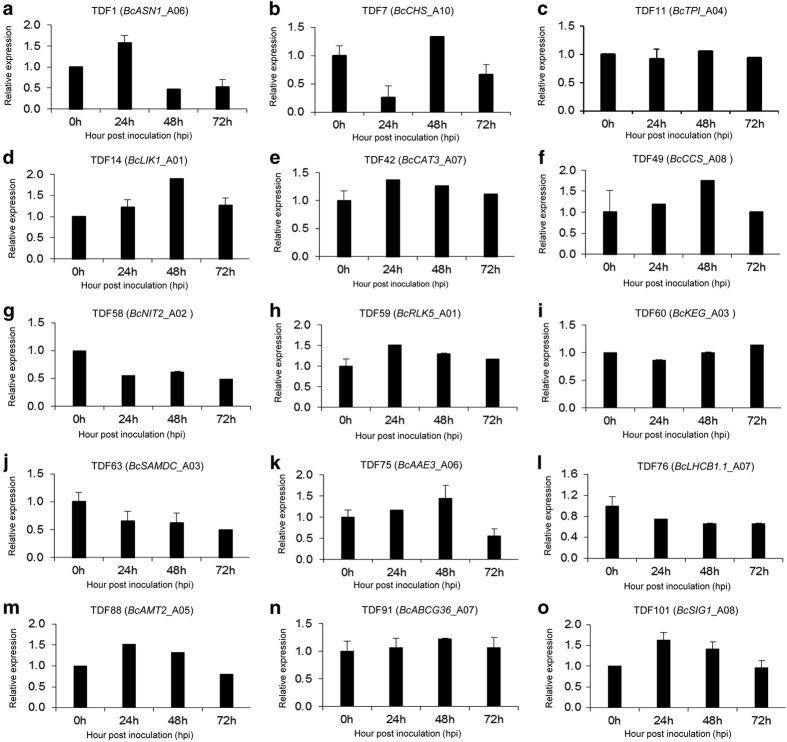
Quantitative real-time PCR analysis of 15 selected genes. (a–o) Both control and treated third leaf of five plants ‘Suzhou Qing’ were harvested and pooled at 0, 24, 48 and 72 h.p.i. The relative expression level for *H. parasitica*-inoculated plants at each time point was calculated as fold of the control plants at 0 using the LOG method. All data were normalized to the *β-actin* gene expression level. Error bars indicate s.d. of the three technical repeats.

To investigate the reliability of cDNA-AFLP for detecting differentially expressed genes, qRT-PCR analysis was carried out for 15 TDFs. These TDFs were selected based on significantly different expression patterns in the time course of the cDNA-AFLP experiment and homology to genes known to have a role in defense, signal transduction, regulation and energy metabolism. Expression patterns of the 15 TDFs in non-heading Chinese cabbage leaves after infection are shown in Figure 3. The same expression pattern was found for each TDF with qRT-PCR analysis as observed in the cDNA-AFLP tests, except for TDF60 (*BcKEG*_A03) and TDF91 (*BcABCG36*_A07). As shown in Figure 3, TDFs that involved in defense (TDF1 (*BcASN1*_A06), TDF7 (*BcCHS*_A10), TDF11 (*BcTPI*_A04) and TDF14 (*BcLIK1*_A01)) had maximum expression at 48 h.p.i., except for TDF1 that had maximum expression peaked at 24 h.p.i. TDFs involved in signal transduction included TDF42 (*BcCAT3*_A07), TDF49 (*BcCCS*_A08), TDF58 (*BcNIT2*_A02), TDF59 (*BcRLK5*_A01), TDF60 (*BcKEG*_A03) and TDF63 (*BcSAMDC*_A03)). Among of them, TDF42, TDF49 and TDF59 had similar expression patterns and peaked at 24 or 48 h.p.i. TDF58 and TDF63 had minimum expression at 72 h.p.i., suggesting that these two genes may be repressed after infection. TDFs involved in energy metabolism included TDF75 (*BcAAE3*_A06), TDF76 (*BcLHCB1.1*_A07) and TDF88 (*BcAMT2*_A05). Among them, TDF75 and TDF88 were expressed highly at 0 h.p.i. compared with other TDFs, and showed maximum expression at 48 and 24 h.p.i., respectively. The results suggested that they may have been involved in the earlier stageinteraction between non-heading Chinese cabbage and *H. Parasitica*. TDF76 expressed very low at the 0 h.p.i. TDF91 (*BcABCG36*_A07) and TDF101 (*BcSIG1*_A08)) were related to regulation. TDF91 was slowly increased after 0 h.p.i. and maximum expression peaked at 48 h.p.i. TDF101 was strongly upregulated at 24 h.p.i. and decreased after 24 h.p.i. These results suggested that the selected TDFs with putative four categories of functions might be triggered rapidly and have an active role during the early incompatible interaction between non-heading Chinese cabbage and *H. parasitica*.

**Figure 4:**
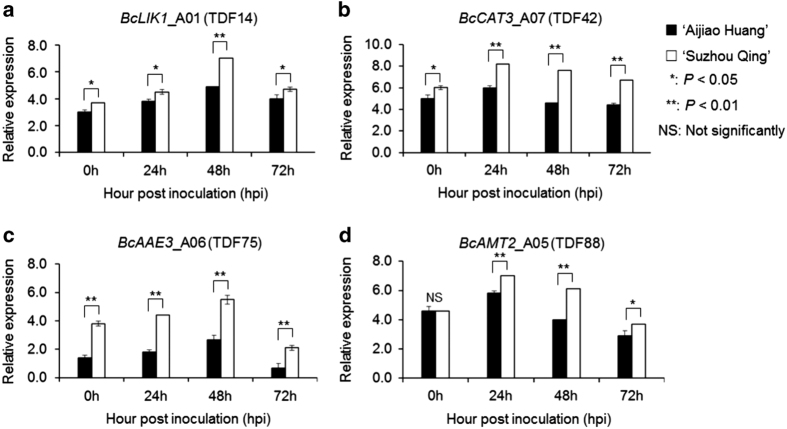
The comparison of partial TDFs (a–d) expression patterns between resistant line and susceptible line. The results show that these four genes has almost the same expression trend between two lines, but the expression of gene in the resistant line ‘Suzhou Qing’ is higher than that of in susceptible line ‘Aijiao Huang’. Both control and treated third leaf of five plants were harvested and pooled at 0, 24, 48 and 72 h.p.i. The gene expression level was calculated using the LOG method. The gene expression level in both lines was compared using LOG value directly. All data were normalized to the *β-actin* gene expression level. Error bars indicate s.d. of the three technical repeats. Asterisks indicate statistically significant differences compared ‘Aijiao Huang’ and ‘Suzhou Qing’ at each TDFs (Student’s *t*-test: *P<0.05; **P<0.01).

Through BLAST searching in the *Arabidopsis* database (http://www.arabidopsis.org/), we found that four of the 15 TDFs were related with fungal resistance. To verify these expectations were related to fungal resistance, we performed a qRT-PCR experiment with a resistant and a susceptible line. Results are shown in Figure 4.

TDF14 (*BcLIK1*_A01) encodes LRR-RLK protein, is involved in regulation of innate immune response, and have a role against pathogens according to the homologous alignment in the *Brassica* database. As shown in Figure 4, gene expression of TDF14 was increased slowly after inoculation and expression peaked at 48 h.p.i. Although both have the same expression trends, the expression of TDF14 in resistant line ‘Suzhou Qing’ is higher than that in susceptible line ‘Aijiao Huang’, especially in 48 h.p.i.

TDF42 (*BcCAT3*_A07) encodes *catalase* and is involved in the regulation of defense. *Catalase* is one of the key enzymes *in vivo* anti-oxidative defense systems, which has a special role in removing the hydrogen peroxide to avoid the body to produce oxidative stress in the process. The expression patterns of TDF42 in two lines were similar to that of TDF14. TDF75 (*BcAAE3*_A06) encodes an *oxalyl-CoA synthetase* and involved in defense response to fungus. The gene expression in resistant line ‘Suzhou Qing’ was much higher than that in susceptible line ‘Aijiao Huang’, with fold change reaching to 100 times at 48 h.p.i.

TDF88 (*BcAMT2*_A05) encodes a high-affinity ammonium transporter and involved in ammonium transmembrane transport and defense response to fungus. The expression of TDF88 was almost the same in 0 h.p.i. in two lines, strongly induced subsequently and maximum expression both peaked at 24 h.p.i. But gene expression of TDF88 in resistant line ‘Suzhou Qing’ was always higher than that in susceptible line ‘Aijiao Huang’. The expression of the genes related with fungal resistance in resistance line were higher than that in susceptible line.

## Discussion

We identified 129 TDFs, of which 121 TDFs were upregulated and eight were down-regulated using cDNA-AFLP.^2,3^ By BLAST searching in the *Brassica* database, these TDFs were classified according to their different functions. The functional categorization showed a complex linkage between proteins encoding by the TDFs. Information obtained from this study may provide a foundation for better understanding defense mechanisms of the non-heading Chinese cabbage with *H. parasitica* incompatible interaction.

### Defense

Our data showed that several transcripts encoding the group of *PR proteins* were differentially expressed in the interaction (Table 2). For example, TDF3 (*β-1, 3-glucanase*) was induced within 24 h.p.i. and its expression peaked at 48 h.p.i. The expression levels of TDF8 (*Hapless 8*), TDF16 (*a thaumatin-like protein*) and TDF28 (*PR 1-like protein*) were induced within 24–72 h.p.i. Previously, we cloned the full length of *β-1, 3-glucanase*, *hapless 8* and *PR 1* genes, and analysed their expression patterns in response to *H. parasitica* infection in ‘Suzhou Qing’ cultivar of non-heading Chinese cabbage.^10^ The accumulations of these two transcripts were upregulated during the infection period, suggesting that these proteins may participate in the defence reaction for non-heading Chinese cabbage against *H. parasitica*. We also found that expression of TDF39 (*ATMT-1*) peaked at 48 h.p.i. In rice and barley, *MT2A* genes were induced by stresses such as drought, cold treatment and wounding or in response to pathogen attacks.^12–14^ Further research revealed that products of homologous *MT* scavenged the reactive oxygen species (ROS), such as OH to H_2_O.^15^ Evidences suggest that the generation of ROS occurs at early stage in the plant–pathogen interaction. Rapid accumulation of ROS causes oxidative burst that results in hypersensitive cell death and cell wall cross-link.^16^ Our data may indicate that the upregulation of *MT2A* in non-heading Chinese cabbage leaves may contribute to ROS accumulation for inducing the hypersensitive response of the plant.

### Signal transduction

Studies suggest that several signal transduction-related proteins are involved in the plant–fungus interactions.^1–3,17^ We also identified many TDFs related to the signal transduction, such as TDF42 (*Catalase 3*), TDF43 (*2-Cys PrxB*), TDF45 (*ATP binding*), TDF50 (*ATRER1A*), TDF58 (*Nitrilase*), TDF48 (*BcCAM3*_A04, *calcium ion binding*) and TDF68 (*WRKY DNA-binding protein*).

*Calcium binding-like proteins* may have a role in signalling pathways against pathogens and wounding.^18^ A number of downstream targets of *calmodulin* (*CaM*), including *nitric oxide synthase*,^19^ barley *MLO protein*,^20^ maize *Ca*^*2+*^*-CaM*^21^ and *transcriptional regulators*,^22^ are involved in plant responses to pathogens. Given that *calcium ion-binding proteins* are important modulators of defence response in pathways for pathogen sensing in plants, the *CAM 3* gene could have a special role as Ca^2+^ sensors during the plant immune response to the fungus *H. Parasitica*.

WRKY proteins are signal transcriptional factors recognizing the TTGAC (C/T) W-box elements in the promoters of a large number of plant defence-related genes.^23^ Many of *WRKY* genes are upregulated particularly in pathogen-infected, wounded or abiotic-treated plants.^24^ In this study, expression of *WRKY DNA-binding protein* peaked at 24 h.p.i., suggesting that the possible role of *WRKYs* is in the regulation of the genes associated with plant defence responses. However, we found that expression pattern of TDF60 (*WRKY* gene) determined by qRT-PCR was inconsistent with that of cDNA-AFLP. The inconsistence may be caused by different paralogues in the genome.

### Regulation

An *ethylene response factor* (*BcERF1*_A01, TDF93; Table 2), a regulator of ethylene responses after pathogen attack in *Arabidopsis*,^25^ may have a key role in the non-heading Chinese cabbage–*H. parasitica* interaction. Previous studies have been demonstrated that ERFs are involved in regulating the expression of the defence-related genes during the disease resistance responses.^26,27^

We found that TDF91 (*BcABCG36*_A07, *ATP-binding cassette g36*) was inhibited after inoculation by cDNA-AFLP analysis. However, its expression was induced weakly at 24 h.p.i., peaked at 48 h.p.i. and decreased weakly at 72 h.p.i. afterwards by qRT-PCR analysis. TDF100 (*BcEDM1*_A09) coding for an enhanced downy mildew 1 homolog was found to be induced during the infection period. Its relationship with the fungi, bacteria and viruses has been identified to be regulators of *R* gene-mediated resistance in other crop species.^28,29^ Recent studies have revealed that *EDM1* homologue gene *SGT1* is required for pathogen-induced disease-associated cell death during both compatible and incompatible interactions in tobacco.^30^

### Energy metabolism

Energy metabolism has an important role in plants–pathogen interaction. The photosynthetic carbon cycle (PCC) is part of the dark reactions of photosynthesis and can be roughly divided into three steps: carboxylation, reduction reaction and regeneration of *RuBP*.^31^ In this study, we found that some TDFs relating to energy metabolism were downregulated, such as TDF76 (*chlorophyll a/b binding protein*), TDF78 (S*IT4 phosphatase-associated family protein*) and TDF82 (*PP2C-related protein*), whereas some were upregulated, such as TDF79 (*receptor like protein 51*), TDF80 (*NADH-ubiquinone oxidoreductase*), TDF83 (*respiratory burst oxidase protein*), TDF85 (*rubisco small subunit 1b*; Table 2). Previous reports have identified that they are involved in PCC cycle, for example, TDF86 (*reduction of transketolase*) inhibited *ribulose-1,5-bisphosphate* regeneration and photosynthesis.^31,32^ These results are consistent with previous report that the expression of energy metabolism-related genes are induced and/or suppressed in photosynthesis during abiotic and biotic stresses.^33,34^ Our results may suggest that PCC cycle could provide protection function in energy metabolism during non-heading Chinese cabbage against *H. parasitica*.

### Protein–protein interaction

A number of genes related to protein–protein interaction were induced after inoculation, such as TDF110 (*BcTPR12*_A07, *tetratricopeptide repeat protein*) and TDF111 (*BcZF_*A01, *zinc-finger family protein*). Of which, the gene expression of *PAT* is induced in the presence of ozone in *Arabidopsis*.^35^ The tryptophan biosynthetic enzymes, including *anthranilate synthase* (*ASA*) and *PAT*, are co-ordinately upregulated at both the messenger RNA and protein level during biotic and abiotic stress.^36^ We found that one of *BcZF* orthologous to *A. thaliana* was induced after inoculation (Table 2). Rizhsky *et al.* speculate that a zinc-finger protein is required for the expression of ascorbate peroxidase, which provides some measure of resistance for plant during oxidative stress.^37^ In this study, fact that pathogen-induced accumulation of these protein–protein interaction-related genes suggested that these genes may be involved in some defence mechanisms against *H. parasitica* indirectly.

Using the cDNA-AFLP method, we also detected several unknown functional genes. Their biological role is still unclear.

In this study, we examined gene expression patterns in an incompatible interaction between non-heading Chinese cabbage ‘Suzhou Qing’ and the downy mildew pathogen. We obtained 129 TDFs with different expression patterns and classified functional categories using cDNA-AFLP. Fifteen TDFs were randomly selected for validation of cDNA-AFLP expression patterns using qRT-PCR. Results showed that reliability of cDNA-AFLP is suitable for detecting differentially expressed genes. Among the 15 TDFs, four TDFs are related with fungal resistance, namely, TDF14 (*BcLIK1*_A01), TDF42 (*BcCAT3*_A07), TDF75 (*BcAAE3*_A06) and TDF88 (*BcAMT2*_A05). We further compared expression patterns in ‘Suzhou Qing’ and ‘Aijiao Huang’ using qRT-PCR. Results showed that the four genes displayed similar expression trend in the two lines. Importantly, the expression of genes in the resistant line is higher than that in susceptible line. These genes expression patterns and their putative functions may provide insight in understanding the non-heading Chinese cabbage–downy mildew incompatible interaction. Our study may also provide a foundation for better understanding molecular mechanisms and can be beneficial in selecting candidate resistance genes for the incompatible interaction between non-heading Chinese cabbage and *H. Parasitica*. Further research is needed to study the comparison between compatible and incompatible interactions to identify novel and common genes that regulate non-heading Chinese cabbage–downy mildew pathosystem.

## Figures and Tables

**Table 1:** Primers and reference sequence used in qRT-PCR analysis

*Functional categories*	*TDFs number*	*Gene*	*Sequence 5′–3′ (forward )*	*Sequence 5′–3′ (reverse)*
D	TDF1	*BcASN1*_A06	TTCCTTCTACGCCTTATG	GAATCAAGACCACCAGAT
D	TDF7	*BcCHS*_A10	TGTGTTCTCTTCATATTGGA	CACTGTCTCTACGGTAAG
D	TDF11	*BcTPI*_A04	CTCAAGTTCCTTCACAAGA	AGTTCACAAGCATCTCAG
D	TDF14	*BcLIK1*_A01	CCTCCTCGTCTCTATCAT	ATTCCAGTTAGTCTTCTTCAA
ST	TDF42	*BcCAT3*_A07	GTCCACACCTACACTCTA	CAACTACCTTAGCCTCTTC
ST	TDF49	*BcCCS*_A08	TTCCTCATCTTCCTCTACTA	CACACTTCATATCCACCAT
ST	TDF58	*BcNIT2*_A02	GCTTCCACTGTCTATAATGA	CTATGCCGAACCTATATCC
ST	TDF59	*BcRLK5*_A01	TCATTCACATTGGTCTTCT	CACATAGTAAGGCGAGAG
ST	TDF60	*BcKEG*_A03	GCCTTACACCGTTACATA	TTATAGCAGCAGCCATAC
ST	TDF63	*BcSAMDC*_A03	GCCTTACACCGTTACATA	TTATAGCAGCAGCCATAC
EM	TDF75	*BcAAE3*_A06	CCTCCGTCAACAACATTA	GGCGTCATACTTCTTCAT
EM	TDF76	*BcLHCB1.1*_A07	GTTGAAGGTGAAGGAGAT	AATGGTCAGCAAGATTCT
EM	TDF88	*BcAMT2*_A05	ACATTAGCGGTATTCTACA	GACACTACATTCCAGACA
R	TDF91	*BcABCG36*_A07	TTGATGCTGATGAAGAGA	GGTGGCTGGATTATACTT
R	TDF101	*BcSIG1*_A08	GTCTTCTTCCTCAGTTCA	TAATCTTCGCCACATCAA
Reference	*β-actin*		GTTGCTATCCAGGCTGTTCT	AGCGTGAGGAAGAGCATAAC

Abbreviations: D, defense; ST, signal transduction; EM, energy metabolism; R, regulation; qRT-PCR, quantitative reverse transcription PCR.

**Table 2:** Homology of obtained TDFs in the non-heading Chinese cabbage–*H. parasitica* interaction

*TDF no.*	*Accession number*	*Length (bp)*	*A. thaliana*	*Gene name in Arabidopsis*	*Gene name in B. rapa*	*Gene name in B. campestris ssp. chinensis*	*Functional categories*	*0 h*	*24 h*	*48 h*	*72 h*
1	AB474661	302	AT3G47340	ASN1	Bra018160	*BcASN1*_A06	D	—	X	x	x
2	AB474690	202	AT1G33970	AT1G33970	Bra028016	*AT1G33970*_A09	D	—	x	X	x
3	AB474678	149	AT3G57260	PR2	Bra014636	*BcBG3*_A04	D	—	x	X	x
4	AB474674	313	AT1G52400	ATBG1	Bra018969	*BcATBG1*_A06	D	x	X	x	x
5	AB474696	127	AT1G55490	CPN60B	Bra011919	*BcCPN60B*_A07	D	—	x	x	x
6	AB474682	297	AT1G02305	AT1G02305	Bra030498	*AT1G02305*_A08	D	—	x	x	x
7	AB474660	221	AT5G13930	CHS	Bra008792	*BcCHS*_A10	D	—	x	X	x
8	AB474679	122	AT5G56250	HAP8	Bra003169	*BcHAP8*_A07	D	—	x	x	x
9	AB474688	80	AT1G08400	AT1G08400	Bra018627	*AT1G08400*_A06	D	—	x	x	x
10	AB474667	228	AT2G16600	ROC3	Bra037296	*BcROC3*_A09	D	—	x	x	x
11	AB474666	441	AT3G55440	TPI	Bra014743	*BcTPI*_A04	D	—	x	X	x
12	AB474665	132	AT1G01040	ASU1	Bra033293	*BcASU1*_A10	D	x	x	X	x
13	AB474676	326	AT2G03820	NMD3	Bra040033	*BcNMD3*_A01	D	—	x	x	x
14	AB474643	304	AT3G14840	LIK1	Bra021579	*BcLIK1*_A01	D	—	x	X	x
15	AB474693	293	AT2G47070	SPL1	Bra041037	*BcSPL1*_Scaffold000403	D	—	x	x	x
16	FJ605478	954	AT1G18250	ATLP-1	Bra025923	*BcATLP-1*_A06	D	—	x	x	x
17	AB474697	223	AT1G60950	FD2	Bra031471	*BcFD2*_A01	D	—	x	x	x
18	AB474681	204	AT1G08540	SIG1	Bra030732	*BcSIG1*_A08	D	—	x	x	x
19	AB474684	160	AT3G60120	BGLU27	Bra004840	*BcBGLU27*_A05	D	—	x	x	x
20	AB474664	383	AT2G41480	PRX25	Bra000228	*BcPRX25*_A03	D	—	x	x	x
21	AB474689	265	AT1G18360	AT1G18360	Bra016558	*AT1G18360*_A08	D	—	x	x	x
22	AB474694	119	AT3G01290	HIR2	Bra039130	*BcHIR2*_A05	D	—	x	x	x
23	AB474671	204	AT1G52120	AT1G52120	Bra018940	*AT1G52120*_A06	D	—	x	x	x
24	AB474685	258	AT5G42870	PAH2	Bra027461	*BcPAH2*_A09	D	—	x	x	x
25	AB474659	100	AT2G23760	BLH4	Bra032147	*BcBLH4*_A04	D	—	x	x	x
26	AB474677	100	AT1G25540	MED25	Bra038087	*BcMED25*_A08	D	—	—	x	x
27	AB474686	67	AT3G14210	ESM1	Bra027359	*BcESM1*_A05	D	x	X	x	x
28	AB474683	126	AT2G14580	PRB1	Bra013123	*BcPR1*_A03	D	—	x	x	x
29	AB474662	138	AT3G27310	PUX1	Bra025265	*BcPUX1*_A06	D	—	—	x	—
30	AB474669	112	AT2G35100	ARAD1	Bra023001	*BcARAD1*_A03	D	—	x	x	x
31	AB474663	255	AT5G63110	HDA6	Bra035858	*BcHDA6*_A09	D	—	—	x	—
32	AB474695	150	AT3G04790	EMB3119	Bra040120	*BcEMB3119*_A01	D	—	X	X	x
33	AB474658	80	AT4G34850	LAP5	Bra011566	*BcLAP5*_A01	D	—	x	x	x
34	AB474687	363	AT5G67360	ARA12	Bra037113	*BcARA12*_A09	D	—	x	x	x
35	AB474668	183	AT5G58070	TIL	Bra006784	*BcTIL*_A03	D	—	x	x	x
36	AB474691	263	AT3G16560	AT3G16560	Bra022166	*AT3G16560*_A05	D	X	x	x	x
37	AB474675	336	AT5G38430	RBCS1B	Bra028181	*BcRBCS1B*_A04	D	—	x	x	X
38	AB439291	556	AT1G54410	ATHIRD11	Bra013206	*BcATHIRD11*_A03	D	x	X	x	x
39	AB439836	528	AT3G09390	ATMT-1	Bra029765	*BcATMT-1*_A05	D	—	x	X	x
40	AB474649	315	AT5G44790	RAN1	Bra025102	*BcRAN1*_A06	D	x	X	x	x
41	AB474717	316	AT5G53300	UBC10	Bra022645	*BcUBC10*_A02	D	—	x	x	x
42	AB474628	266	AT1G20620	CAT3	Bra012238	*BcCAT3*_A07	ST	—	X	X	x
43	AB495004	917	AT5G06290	2CPB	Bra009181	*Bc2CPB*_A10	ST	x	x	X	x
44	AB474632	843	AT1G12520	CCS	Bra016768	*BcCCS*_A08	ST	x	x	x	x
45	AB474627	239	AT5G56500	CPN60BETA3	Bra028922	*BcCPN60BETA3*_A03	ST	—	x	X	x
46	AB474645	121	AT5G18020	SAUR20	Bra026598	*BcSAUR20*_A02	ST	—	x	x	x
47	AB474656	264	AT4G28080	AT4G28080	Bra024230	*AT4G28080*_A03	ST	—	X	x	x
48	AB474629	140	AT3G56800	CAM3	Bra014671	*BcCAM3*_A04	ST	—	X	x	x
49	AB474632	843	AT1G12520	CCS	Bra016768	*BcCCS*_A08	ST	—	—	X	x
50	AB474637	266	AT4G39220	ATRER1A	Bra010695	*BcATRER1A*_A08	ST	—	—	x	x
51	AB474646	136	AT2G33150	PKT3	Bra005522	*BcPKT3*_A05	ST	—	x	X	x
52	AB474648	128	AT5G13650	SVR3	Bra008811	*BcSVR3*_A10	ST	—	x	X	x
53	AB474647	252	AT2G31060	EMB2785	Bra021694	*BcEMB2785*_A04	ST	—	X	X	x
54	AB474635	638	AT4G00700	AT4G00700	Bra000963	*AT4G00700*_A03	ST	—	X	X	x
55	AB474640	95	AT2G01400	AT2G01400	Bra026670	*BcAT2G01400*_A02	ST	—	—	x	—
56	AB474652	160	AT4G26240	AT4G26240	Bra019116	*AT4G26240*_A03	ST	—	x	x	x
57	AB474657	224	AT3G63070	HULK3	Bra040426	*BcHULK3*_A04	ST	—	—	x	X
58	AB474655	186	AT3G44300	NIT2	Bra023598	*BcNIT2*_A02	ST	X	x	x	x
59	AB474634	267	AT4G28490	RLK5	Bra011033	*BcRLK5*_A01	ST	—	X	x	x
60	AB474641	226	AT5G13530	KEG	Bra006205	*BcKEG*_A03	ST	X	x	x	x
61	AB474633	231	AT2G22260	ALKBH2	Bra038536	*BcALKBH2*_A09	ST	—	x	x	—
62	AB474639	166	AT4G13940	MEE58	Bra032750	*BcMEE58*_A04	ST	x	x	X	x
63	AB474638	306	AT3G02470	SAMDC	Bra001046	*BcSAMDC*_A03	ST	X	x	x	x
64	AB474651	405	AT3G55800	SBPASE	Bra007192	*BcSBPASE*_A09	ST	—	—	x	x
65	AB474636	736	AT4G32200	ASY2	Bra039766	*BcASY2*_A01	ST	—	—	x	x
66	AB474630	369	AT5G16070	AT5G16070	Bra006338	*AT5G16070*_A03	ST	—	X	X	x
67	AB474654	236	AT4G21450	AT4G21450	Bra013528	*AT4G21450*_A01	ST	—	X	x	x
68	AB474650	180	AT4G31800	WRKY18	Bra023983	*BcWRKY18*_A03	ST	x	X	x	x
69	AB474714	291	AT3G22650	AT3G22650	Bra005858	*AT3G22650*_A03	ST	—	x	x	x
70	AB474715	199	AT1G47210	CYCA3;2	Bra040753	*BcCYCA3;2*_Scaffold000249	ST	—	X	X	x
71	AB474720	188	AT2G33800	EMB3113	Bra021879	*BcEMB3113*_A04	ST	—	x	x	x
72	AB474716	126	AT5G42220	AT5G42220	Bra027974	*AT5G42220*_A09	ST	—	—	x	x
73	AB474644	121	AT2G33620	AHL10	Bra021865	*BcAHL10*_A04	ST	—	x	x	x
74	AB474713	129	AT3G15290	AT3G15290	Bra027263	*AT3G15290*_A05	EM	—	X	X	x
75	AB474703	127	AT3G48990	AAE3	Bra018019	*BcAAE3*_A06	EM	—	x	X	x
76	AB474710	384	AT1G29920	LHCB1.1	Bra010807	*BcLHCB1.1*_A07	EM	X	x	x	x
77	AB474711	405	AT1G29920	CAB2	Bra010807	*BcCAB2*_A08	EM	x	x	x	x
78	AB474704	150	AT3G45190	AT3G45190	Bra038298	*AT3G45190*_A10	EM	X	x	x	x
79	AB474705	427	AT4G18760	RLP51	Bra040730	*BcRLP51*_A06	EM	—	x	x	—
80	AB474702	150	AT5G18800	AT5G18800	Bra002186	*AT5G18800*_A10	EM	—	x	x	x
81	AB474709	266	AT4G03280	PGR1	Bra000837	*BcPGR1*_A03	EM	—	x	—	x
82	AB474708	75	AT3G16560	AT3G16560	Bra022166	*AT3G16560*_A05	EM	X	x	x	x
83	AB474706	222	AT5G47910	RBOHD	Bra020724	*BcRBOHD*_A02	EM	—	x	x	x
84	AB474712	239	AT5G38430	RBCS1B	Bra028181	*BcRBCS1B*_A04	EM	—	X	x	x
85	AB439290	733	AT5G38430	RBCS1B	Bra028174	*BcRBCS1B*_A04	EM	—	X	x	x
86	AB474707	53	AT3G60750	TKL1	Bra007555	*BcTKL1*_A09	EM	—	—	x	x
87	AB474626	190	AT5G36880	ACS	Bra030286	*BcACS*_A04	EM	—	x	X	x
88	AB474613	210	AT2G38290	AMT2	Bra005125	*BcAMT2*_A05	EM	—	X	x	x
89	AB474614	169	AT2G33150	PKT3	Bra022927	*BcPKT3*_A03	R	—	—	x	x
90	AB474609	106	AT4G00810	AT4G00810	Bra037409	*AT4G00810*_A09	R	—	x	—	—
91	AB474616	349	AT1G59870	ABCG36	Bra003527	*BcABCG36*_A07	R	X	x	x	x
92	AB474621	144	AT4G34970	ADF9	Bra017683	*BcADF9*_A03	R	—	x	x	x
93	AB474610	140	AT3G23240	ERF1	Bra023744	*BcERF1*_A01	R	—	x	x	x
94	AB474624	310	AT3G55360	GLH6	Bra007154	*BcGLH6*_A09	R	—	x	x	x
95	AB474619	211	AT4G27640	AT4G27640	Bra026329	*AT4G27640*_A01	R	—	x	x	x
96	AB474612	119	AT1G35160	14-3-3PHI	Bra028068	*Bc14-3-3PHI*_A09	R	—	x	X	X
97	AB439837	1062	AT1G01050	PPA1	Bra033292	*BcPPA1*_A10	R	x	—	x	x
98	AB474615	230	AT5G14400	CYP724A1	Bra023464	*BcCYP724A1*_A02	R	—	x	x	x
99	AB474718	200	AT3G62310	AT3G62310	Bra007671	*AT3G62310*_A09	R	—	X	X	x
100	AB495003	1418	AT4G11260	EDM1	Bra035239	*BcEDM1*_A09	R	x	X	x	x
101	AB474611	393	AT1G08540	SIG1	Bra030732	*BcSIG1*_A08	R	—	X	X	x
102	AB474719	173	AT5G02500	AT-HSC70-1	Bra009584	*BcAT-HSC70-1*_A10	R	—	x	x	x
103	AB474622	429	AT5G64940	ATH13	Bra024339	*BcATH13*_A06	R	—	X	x	x
104	AB474623	218	AT5G54770	THI4	Bra022742	*BcTHI4*_A02	R	—	X	x	x
105	AB474625	150	AT3G55360	GLH6	Bra007154	*BcGLH6*_A09	R	x	X	X	X
106	AB474618	220	AT1G65550	AT1G65550	Bra036544	*AT1G65550*_A09	R	x	x	x	x
107	AB474620	183	AT1G55620	CLCF	Bra038007	*BcRRN23S.2*_A06	R	—	x	x	x
108	AB474699	478	AT3G32195	AT3G32195	Bra007659	*AT3G32195*_A09	PPI	—	x	x	x
109	AB474701	448	AT4G00700	AT4G00700	Bra000963	*AT4G00700*_A03	PPI	—	x	x	x
110	AB474698	180	AT1G78120	TPR12	Bra015628	*BcTPR12*_A07	PPI	—	x	x	x
111	AB474700	423	AT4G28270	ZF	Bra026271	*BcZF*_A01	PPI	—	x	x	x
112	AB474727	308	AT5G53370	PMEPCRF	Bra003062	*BcPMEPCRF*_A10	O	x	—	x	x
113	AB474721	113	AT3G62770	ATG18A	Bra003509	*ATG18A*_A07	O	—	X	X	x
114	AB474726	583	AT1G19480	AT1G19480	Bra025741	*AT1G19480*_A06	O	x	X	x	x
115	AB474723	175	AT1G62970	AT1G62970	Bra027007	*AT1G62970*_A09	O	—	x	X	x
116	AB474730	340	AT4G34640	SQS1	Bra011548	*BcSQS1*_A01	O	x	x	x	—
117	AB474741	209	AT3G13720	PRA1.F3	Bra027406	*BcPRA1.F3*_A05	Un	—	X	x	x
118	AB474742	149	AT1G44191	AT1G44191	Bra010149	*AT1G44191*_A06	Un	—	—	x	x
119	AB474745	149	AT3G13080	MRP3	Bra039368	*BcMRP3*_Scaffold000164	Un	—	—	—	x
120	AB474733	126	AT1G14740	TTA1	Bra026192	*BcTTA1*_A06	Un	—	X	x	x
121	AB474737	205	AT3G29075	AT3G29075	Bra025663	*AT3G29075*_A04	Un	x	X	x	x
122	AB474740	149	AT3G13080	MRP3	Bra039368	*BcMRP3*_A06	Un	x	X	x	x
123	AB474736	121	AT5G42050	AT5G42050	Bra025450	*AT5G42050*_A04	Un	—	X	x	x
124	AB474731	130	AT5G09805	IDL3	Bra009082	*BcIDL3*_A10	Un	x	—	X	—
125	AB474738	191	AT1G70900	AT1G70900	Bra016176	*AT1G70900*_A07	Un	x	X	x	x
126	AB474735	239	AT3G29075	AT3G29075	Bra025663	*AT3G29075*_A04	Un	x	—	—	x
127	AB474739	77	AT3G49601	AT3G49601	Bra017971	*AT3G49601*_A06	Un	—	X	X	—
128	AB474732	398	AT4G19430	AT4G19430	Bra013396	*AT4G19430*_A01	Un	—	—	X	—
129	AB474746	301	AT5G44790	RAN1	Bra025102	*BcRAN1*_A06	Un	—	x	x	—

Abbreviations: D, defense; EM, energy metabolism; O, others; PPI, protein-protein interaction; R, regulation; ST, signal transduction; Un, unknown. Legend: (X,X) Different signal intensity in cDNA-AFLP analysis (6% polyacrylamide gel). The bigger legend "X", the more signal intensity.
